# Supplementary material for: Cardiomyocyte Perm1 sustains cardiac bioenergetic reserve and exercise capacity
Source: bioRxiv. 2026 Oct 10:2026.10.03.753324. Preprint. [Version 1] doi: 10.64898/2026.10.03.753324 (PMC13692043; doi:10.64898/2026.10.03.753324)
Supplement: Supplementary file 1 [file media-1.pdf]

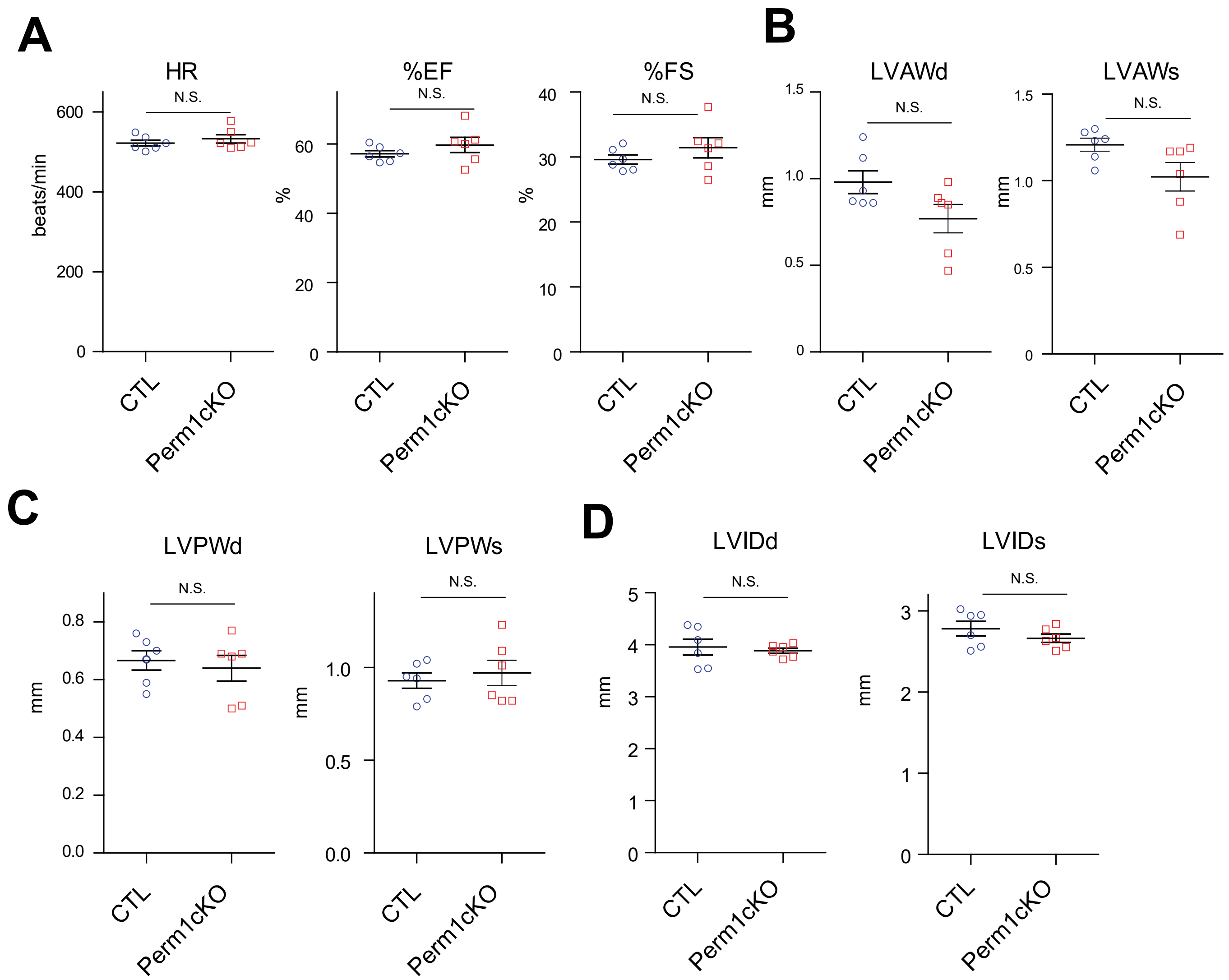

Supplemental Fig. S1

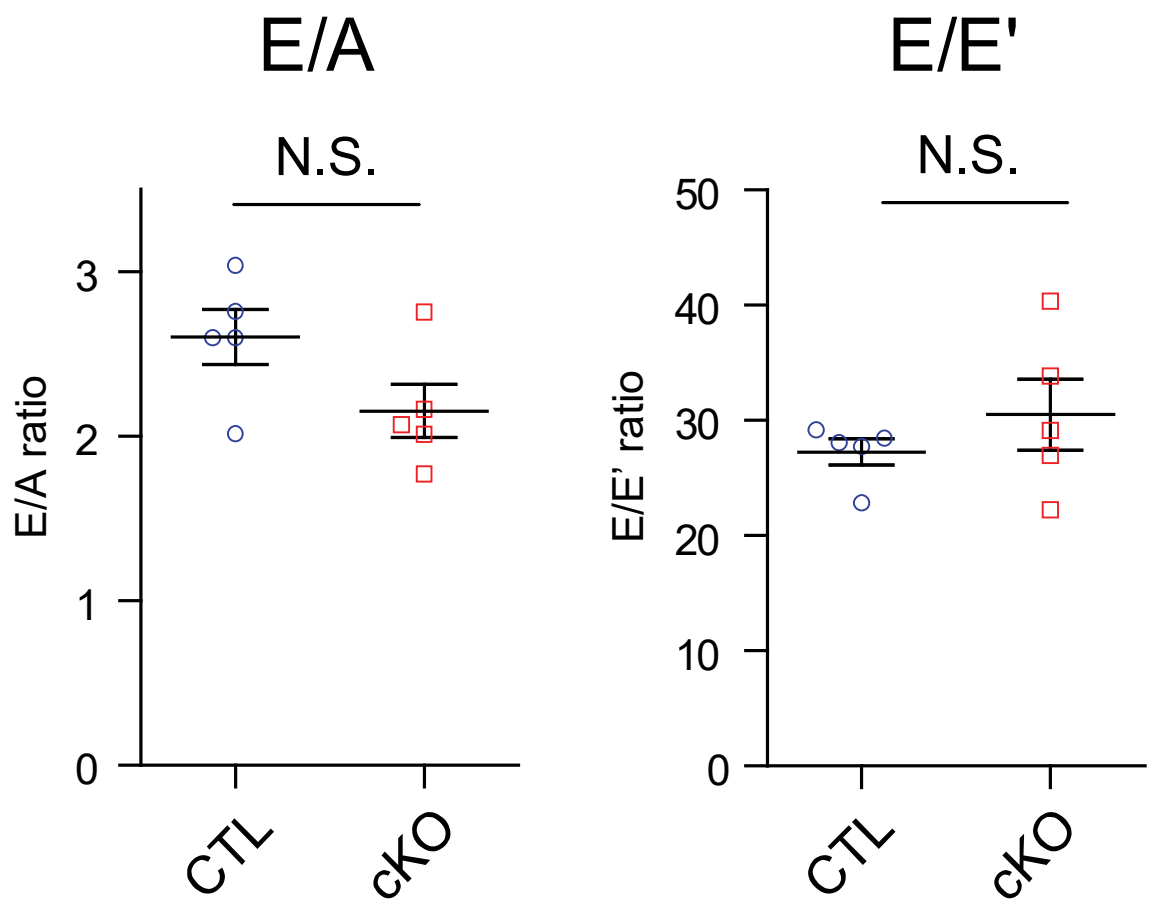

Supplemental Fig. S2

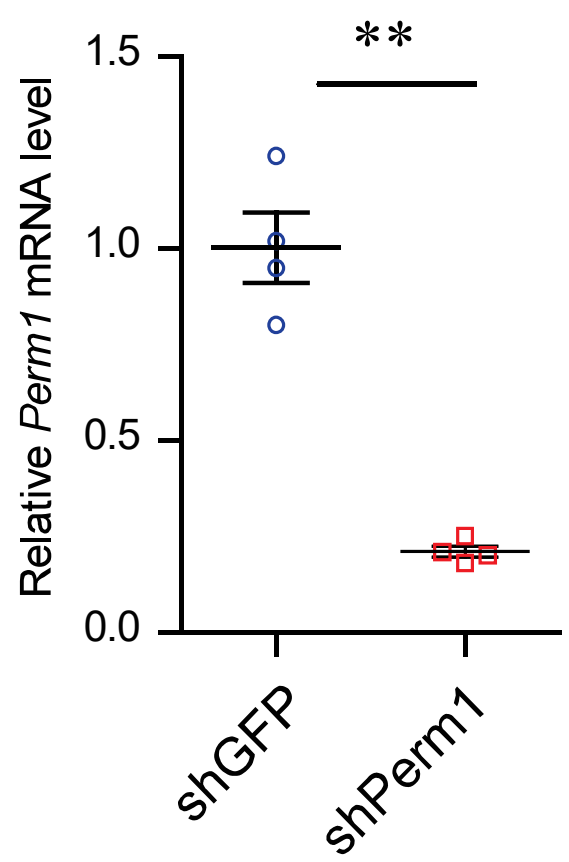

Supplemental Fig. S3
