# Supplementary material for: Cardiomyocyte Perm1 sustains cardiac bioenergetic reserve and exercise capacity
Source: bioRxiv. 2026 Oct 10:2026.10.03.753324. Preprint. [Version 1] doi: 10.64898/2026.10.03.753324 (PMC13692043; doi:10.64898/2026.10.03.753324)
Supplement: Supplementary file 2 [file media-2.pdf]

Supplemental Table 1. Oligonucleotide sequences used in this study.		
**Name**		
**CRISPR/Cas9 reagents for generating the Perm1 floxed allele**		
Perm1-L crRNA	AGAUAGGAGGACAGACCCUGGUUUUAG AGCUAUGCUGUUUUG	
Perm1-R crRNA	CACUGUCCUAGAUGCCAUCAGUUUUAG AGCUAUGCUGUUUUG	
Perm1-L donor ssDNA	ATTGGGCAAAATGGGGCCTTGGTTAGC AGAGCAGGAATAGATAGGAGGACAGAC CATAACTTCGTATAGCATACATTATAC GAAGTTATCTGAGGACAGCCAGAATGG ACTGCTGGAGTTCAGGGAGTTCTTATT TAAGTTCTA	
Perm1-R donor ssDNA	AAGCCAGACACTTCAGGGGTTTGGGCC TTGTCACTGACCACTGTCCTAGATGCC AATAACTTCGTATAGCATACATTATAC GAAGTTATTCAGGGACTTTCCTCTAGC TTTTAAGGGCCTGGCACTAGAGGCCCA CTCACACTC	
**Name**	**Forward / Sense (5'→3')**	**Reverse / Antisense (5'→3')**
**Genotyping PCR primers**		
Perm1 floxed allele (5' loxP)	GAGAGGAGAGAAAGGTTGATAGG	GAATCTATAGTGTCCAGCAGGTGA
Perm1 floxed allele (3' loxP)	AAGAATAATCTCCCACGCTATC	AGAGATCTTTTTCTCACACTG
Cre transgene	TGCCTGCATTACCGGTCGATGC	CCATGAGTGAACGAACCTGGTCG
**Quantitative RT-PCR primers**		
Perm1	TGCCTATTTCCATCCCTGAG	GGCACATGTCGTTTTGACTG
Pgc-1α (Ppargc1a)	CCTCATAAAGCCAACCAAGATAA	GTTTAGTCTTCCTTTCCTCGTGTC
Pgc-1β (Ppargc1b)	CTCCTCCTGGCCACATCCT	ACACAAGGCCGCTGACTTC
Errα (Esrra)	GGAGGACGGCAGAAGTACAAA	GCGACACCAGAGCGTTCAC
Errβ (Esrrb)	GGAATGCTAAAACGGGACTGA	TGATGAAGGAGCCGCAACTA
Errγ (Esrrg)	TCCCCGACAGTGACATCAAA	GTGTGGAGAAGCCTGGAATA
Pparα (Ppara)	AAGGCTATCCCAGGCTTTGC	TTTAGAAGGCCAGGCCGATCTC
Pparδ (Ppard)	GTATGCGCATGGGACTCAC	GTCTGAGCGCAGATGGACT
Tfam	caaaggatgattcggctcag	aagctgaatatatgcctgcttttc
Nrf-1	CCACGTTGGATGAGTACACG	CTGAGCCTGGGTCATTTTGT
Nrf-2 (Gabpa)	CCGCTACACCGACTACGATT	ACCTTCATCACCAACCCAAG
Mfn2	GAAAGTCGAGGCTCTGGATTCA	GACTGGGTGCTTTCCTTCTCTCT
Glut4 (Slc2a4)	GATTCTGCTGCCCTTCTGTCC	CGCTCTCTCTCCAACTTCCG
Ldhb	CCAGCAGGAGGGGGAGAGT	TGCTTAGGTAGCCCGCTCAG
Pdk4	GTTCCTTCACACCTTCACCAC	CCTCCTCGGTCAGAAATCTTG
Lpl	CCTGATGACGCTGATTTTGTAGA	CAGCAGGGAGTCAATGAAGAGA
Fabp3	AAGTGGAACGGGCAGGAGA	GAGGAGCGGGCGGTCAG
Cpt1b	TCTAGGCAATGCCGTTCAC	GAGCACATGGGCACCATAC
Lcad (Acadl)	TCTTTTCCTCGGAGCATGACA	GACCTCTCTACTCACTTCTCCAG
Atgl	CCTGATGACCACCCTTTCCAAC	ACCCGTCTGCTCTTTCATCCAC

Ckmt2	TGTCTGAGATGACGGAGCAG	CCTGGTGTGGTCTTCCTCAT
Idh3a	AGGACTGATTGGAGGTCTTGG	ATCACAGCACTAAGCAGGAGG
Cox7a1	GACAATGACCTCCCAGTACAC	GCCCAGCCCAAGCAGTATAAG
Anf (Nppa)	TACAGTGCGGTGTCCAACACAG	TGCTTCCTCAGTCTGCTCACTC
Bnp (Nppb)	CTGAAGGTGCTGTCCCAGAT	CCTTGGTCCTTCAAGAGCTG
36B4 (Rplp0)	CTGTGCCAGCTCAGAACACTG	TGATCAGCCCGAAGGAGAAG
**Primers for mitochondrial DNA**		
mt-CoxII (mt-Co2)	TCTCCCCTCTCTACGCATTCTA	ACGGATTGGAAGTTCTATTGGC
Dio3	GGAGGTTGTCCGACCTGAT	GTCCCTTGTGCGTAGTCGAG
