# Supplementary material for: Cardiomyocyte Perm1 sustains cardiac bioenergetic reserve and exercise capacity
Source: bioRxiv. 2026 Oct 10:2026.10.03.753324. Preprint. [Version 1] doi: 10.64898/2026.10.03.753324 (PMC13692043; doi:10.64898/2026.10.03.753324)
Supplement: Supplementary file 3 [file media-3.pdf]

Supplemental Table 2. Antibodies used in this study.

Target	Source	Catalog No.
**Target protein**		
PERM1	Sigma-Aldrich	HPA031711
**MICOS complex and mitochondrial dynamics**		
Mitofilin / IMMT (MIC60)	Cell Signaling Technology	30671
CHCHD3 (MIC19)	Novus Biologicals	NBP1-83656
CHCHD6 (MIC25)	Novus Biologicals	NBP1-91779
DRP1	Cell Signaling Technology	8570
OPA1	Cell Signaling Technology	80471
**Oxidative phosphorylation**		
Total OXPHOS rodent cocktail (CI–CV)	Abcam	ab110413
SDHA (Complex II)	Cell Signaling Technology	11998
**Metabolic enzymes**		
PDH (pyruvate dehydrogenase)	Cell Signaling Technology	2784
PDK4	Nuvos Biologicals	NBP1-07047
CKMT2	Proteintech	13207-1-AP
Hexokinase I (HKI)	Cell Signaling Technology	2024
Hexokinase II (HKII)	Cell Signaling Technology	2867
CPT1b	Cell Signaling Technology	41803
LCAD (ACADL)	Proteintech	17442-1-AP
ATGL (PNPLA2)	Cell Signaling Technology	2138
**Loading control**		
GAPDH	Cell Signaling Technology	2118
**Secondary antibodies**		
Anti-rabbit IgG, HRP-linked	Cell Signaling Technology	7074
Anti-mouse IgG, HRP-linked	Cell Signaling Technology	7076
